# *Ex Vivo* and *In Vivo* Neuroprotection Induced by Argon When Given after an Excitotoxic or Ischemic Insult

**DOI:** 10.1371/journal.pone.0030934

**Published:** 2012-02-22

**Authors:** Hélène N. David, Benoît Haelewyn, Mickael Degoulet, Denis G. Colomb, Jean-Jacques Risso, Jacques H. Abraini

**Affiliations:** 1 Université Laval, Centre de Recherche – Centre Hospitalier Affilié Universitaire Hôtel-Dieu de Lévis, Lévis, Québec, Canada; 2 Université de Caen Basse Normandie, CURB, Caen, France; 3 Université de Caen Basse Normandie, UMR 6232, Caen, France; 4 CNRS, UMR 6232, Caen, France; 5 Navy Experimental Diving Unit, Panama City, Florida, United States of America; 6 Institut de Recherche Biomédicale des Armées, antenne Toulon, France; 7 Université Laval, Centre de Recherche – Institut Universitaire en Santé Mentale de Québec, Québec, Québec, Canada; Massachusetts General Hospital/Harvard Medical School, United States of America

## Abstract

*In vitro* studies have well established the neuroprotective action of the noble gas argon. However, only limited data from *in vivo* models are available, and particularly whether postexcitotoxic or postischemic argon can provide neuroprotection *in vivo* still remains to be demonstrated. Here, we investigated the possible neuroprotective effect of postexcitotoxic-postischemic argon both *ex vivo* in acute brain slices subjected to ischemia in the form of oxygen and glucose deprivation (OGD), and *in vivo* in rats subjected to an intrastriatal injection of N-methyl-D-aspartate (NMDA) or to the occlusion of middle-cerebral artery (MCAO). We show that postexcitotoxic-postischemic argon reduces OGD-induced cell injury in brain slices, and further reduces NMDA-induced brain damage and MCAO-induced cortical brain damage in rats. Contrasting with its beneficial effect at the cortical level, we show that postischemic argon increases MCAO-induced subcortical brain damage and provides no improvement of neurologic outcome as compared to control animals. These results extend previous data on the neuroprotective action of argon. Particularly, taken together with previous *in vivo* data that have shown that intraischemic argon has neuroprotective action at both the cortical and subcortical level, our findings on postischemic argon suggest that this noble gas could be administered during but not after ischemia, *i.e.* before but not after reperfusion has occurred, in order to provide cortical neuroprotection and to avoid increasing subcortical brain damage. Also, the effects of argon are discussed as regards to the oxygen-like chemical, pharmacological, and physical properties of argon.

## Introduction

Acute brain ischemia is primarily caused by a disruption of cerebral blood flow through thromboembolism that leads to an oxygen and glucose deprivation, excessive glutamate release and subsequent postsynaptic overstimulation of glutamate receptors, a process known to be critical in ischemia-induced neuronal death [1]–[3]. Over the past 10 years, a series of in vitro and *in vivo* studies in models of hypoxic/ischemic insults has demonstrated the neuroprotective potential of some inert gases, among which xenon has been identified as the most promising agent [4]–[13]. However, the major obstacle to the widespread clinical use of xenon is its scarceness and excessive cost of production. Interestingly argon, which is a cost-efficient and easily available gas with no narcotic and anesthetic action at normal atmospheric pressure, has been also shown to provide organoprotection and neuroprotection against hypoxic-ischemic insults [14]–[18] (but see reference [19] for discrepant results). Recent *in vivo* studies have further shown that intraischemic argon at 50 vol% (with the remainder being 50 vol% oxygen) provides both cortical and subcortical neuroprotection in rats subjected to transient middle cerebral artery occlusion (MCAO) [18]. However, despite this latter study, our knowledge on the ability of argon to provide neuroprotection in acute ischemic stroke still remains limited and should be augmented to evaluate the actual neuroprotective potential of this gas. Particularly, whether argon could provide neuroprotection when given after ischemia still remained unknown. This latter point is not trivial since previous data have shown the critical importance of the time at which inert gases are administered, during or after ischemia [7], [20], to obtain neuroprotection.

Therefore, in the present report, in order to assess thoroughly the neuroprotective potential of argon and to determine whether argon could be a cost-efficient alternative to xenon, we studied the neuroprotective effect of postischemic argon in vitro in brain slices exposed to ischemia in the form of oxygen and glucose deprivation (OGD) and *in vivo* in rats subjected to transient MCAO-induced ischemia or NMDA-induced excitotoxic insult.

## Results

### Effect of argon on OGD-induced cell injury *in vitro*

**Figure 1:**
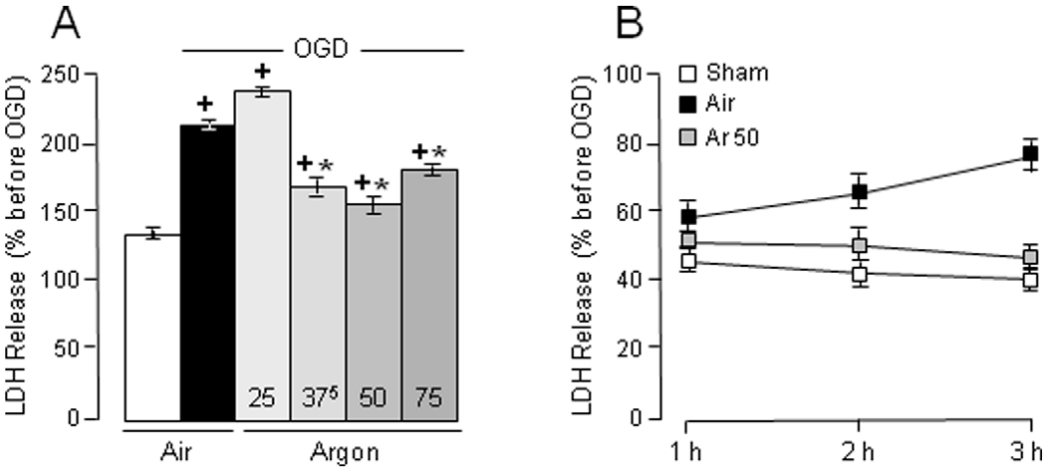
Reduction by argon (Ar) of OGD-induced LDH release. (**A**) Concentration-response effects of argon (Ar) on OGD-induced LDH release (n = 23–27 per group). Exposure to OGD led to an increase in LDH release. Argon of 37.5 vol% to 75 vol% significantly reduced the increase in LDH release induced by OGD. (**B**) Time-course effects of OGD-induced cell injury and argon-induced neuroprotection. Cell injury induced by OGD as well as neuroprotection by argon increased as a function of time during the 3-h “reperfusion” period. Data are expressed as mean ± SEM. * *P*<0.05 *vs* OGD control slices; **^+^** *P*<0.05 *vs* sham slices.

First, we investigated the effect of postischemic argon on neuronal injury induced by OGD in acute brain slices. Cell injury was assessed by the release of OGD-induced lactate dehydrogenase (LDH). Exposure to OGD led to a sustained increase of LDH release compared to sham slices (*P*<0.0001; Fig. 1A,B). Argon given after OGD, *i.e.* during the postischemic “reperfusion” period, led to significant changes in OGD-induced LDH release (*P*<0.0001), so that LDH levels with argon of 37.5 vol% to 75 vol% were lower than those recorded in OGD control slices exposed to nitrogen (*P*<0.01−0.0001; Fig. 1A). Maximal neuroprotection occurred with argon at 50 vol%, and increased with time (Fig. 1B).

### Effect of argon on NMDA-induced neuronal death *in vivo*

**Figure 2:**
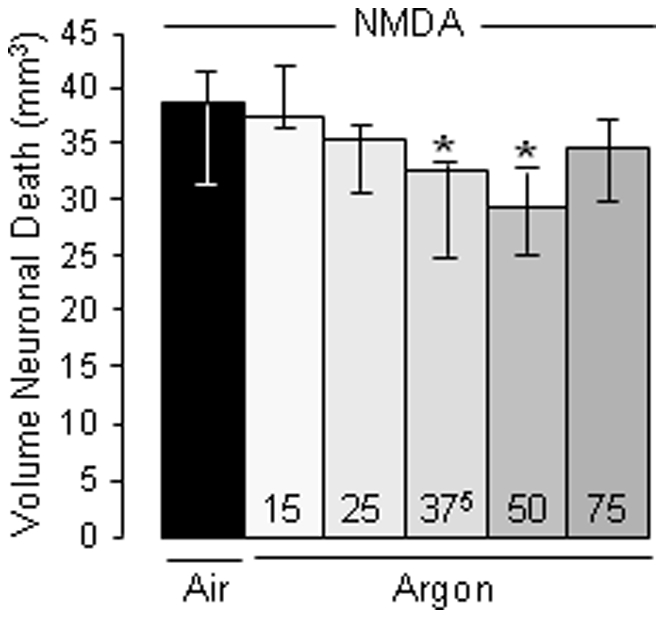
Concentration-response effects of argon (Ar) on NMDA-induced neuronal death (n = 7–11 per group). Injection of NMDA led to significant neuronal death. Sham rats had no brain damage data not shown). Rats treated with argon at 37.5 vol% and 50 vol% had a significant reduction of neuronal death as compared to NMDA control animals treated with medical air. Data are expressed as the median value and 25–75^th^ percentiles * *P*<0.05 *vs* NMDA control rats treated with medical air.

Next, because postsynaptic overactivation of NMDA receptors plays a critical role in ischemic brain damage, we investigated the concentration-response effects of argon on NMDA-induced neuronal death when given 1 h after injection. Intrastriatal injection of NMDA led to significant brain damage compared to sham rats injected with saline (*P*<0.001). Argon of 15 vol% to 75 vol% reduced NMDA-induced brain damage (*P*<0.05), so that rats treated with argon at 37.5 vol% or 50 vol% had reduced brain damage compared to control animals treated with medical air (*P*<0.02–0.05; Fig. 2).

### Effects of argon on MCAO-induced brain damage and behavioral motor alterations

**Figure 3:**
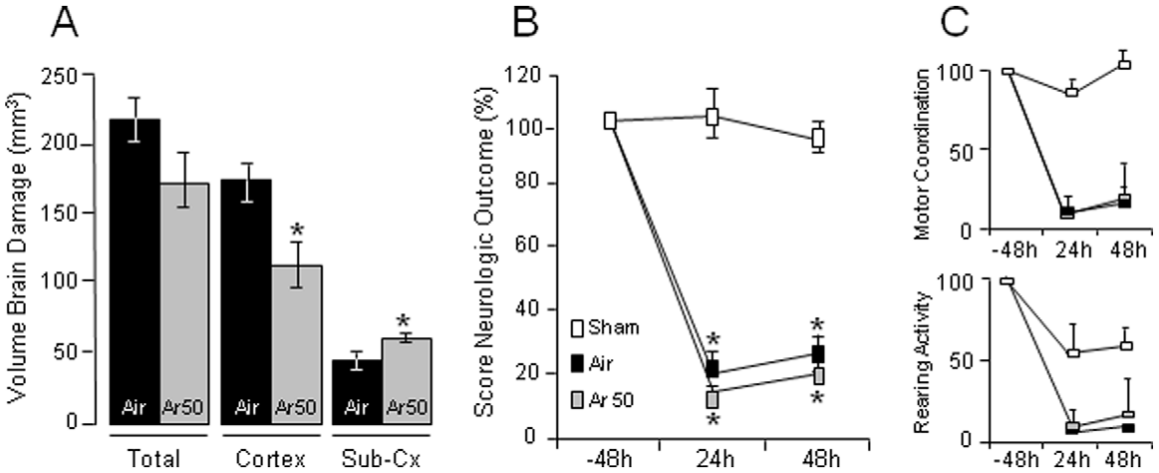
Effects of argon (Ar) at 50 vol% on MCAO-induced brain damage and behavioral motor deficits (n = 14 per group). (**A**) Control rats subjected to MCAO (treated with medical air) exhibited significant cortical and subcortical brain damage. Sham rats had no brain damage (data not shown). Argon at 50 vol% produces cortical neuroprotection, but did increase subcortical brain damage. (**B**) MCAO-induced global score of neurologic outcome. Control rats (line with black boxes), but not sham rats (line with white boxes), exhibited neurological deficits. Rats treated with argon at 50 vol% (line with gray boxes) showed no improvement of neurologic deficits as compared to control rats treated with medical air. (**C**) Scores of motor coordination and rearing activity in sham rats, control animals treated with medical air, and rats treated with argon at 50 vol%. Data are expressed as mean ± SEM. * *P*<0.05 *vs* MCAO control rats treated with medical air; ^+^ *P*<0.05 *vs* sham rats.

Next, based on the *ex vivo* and *in vivo* findings above that indicate that maximal neuroprotection by postinsult argon occurs at 50 vol%, we studied the effects of postischemic argon at 50 vol% on MCAO-induced brain damage and neurologic deficits. When given 1 h after reperfusion, *i.e.* 2 h after MCAO induction, argon reduced cortical volumes of brain damage by ∼35% (*P*<0.02; Fig. 3A) but increased subcortical brain damage by ∼35% (*P*<0.02; Fig. 3A) compared to control rats treated with air. At the behavioral motor level, MCAO rats treated with argon still exhibited a decreased score of neurologic outcome on day 1 (*P*<0.0001) and day 2 (*P*<0.0001) postischemia as compared to sham rats (Fig. 3B) due to marked deficits in motor coordination and rearing activity similar to those of control rats treated with medical air (Fig. 3C; *P*<0.0001). Also, argon did increase the rats' body temperature by 1.1°C (*P*<0.0001), but had no effect on other physiological parameters (Table 1).

**Table 1:** Physiological parameters in control rats treated with medical air and rats treated with argon at 50 vol% before and after MCAO-induced ischemia.

	PaO2	PaCO2	SaO2	Arterial pH	Temp.
Medical Air					
Before	94.3±5.1	32.5±4.8	97.2±0.6	7.41±0.04	37.3±0.3
After	95.0±22.2	34.0±8.0	96.7±0.2	7.40±0.05	37.5±0.2
Argon 50 vol%					
Before	92.6±13.3	32.0±2.2	97.2±0.6	7.42±0.01	37.2±0.4
After	91.5±3.9	32.5±1.7	97.2±0.3	7.42±0.01	38.5±0.6*

Values are expressed as mean ± SD. PaO_2_: arterial oxygen partial pressure in mmHg; PaCO_2_: arterial carbon dioxide partial pressure in mmHg; SaO_2_: arterial hemoglobin saturation of oxygen in %; Temp.: rats' body temperature in °C. Administration of argon at 50 vol% significantly decreases the rats' body temperature by 1.1°C.

*P<0.0001.

## Discussion

The present study shows that argon given after an excitotoxic or ischemic insult reduces cell injury induced by OGD *ex vivo*, subcortical (mainly striatal) neuronal death induced by NMDA injection, and cortical brain damage induced by MCAO. In contrast with these beneficial effects, postinsult argon did increase subcortical brain damage and failed to reduce neurologic deficits induced by MCAO.

Argon possesses oxygen-like properties that could explain at least partly its neuroprotective action. As reported by Semenov, the Nobel Prize Laureate of chemistry in 1956 with Sir Cyril Norman Hinshelwood for their work about branched chain reactions, argon that is incapable of chemical reactions behaves however as a sort of catalyst for some of them by producing a kind of oxygen synergy [21]. In that way, physiological studies have clearly demonstrated that argon at 50 vol% allowed rats exposed to hypoxic conditions incompatible with life to adapt and maintain their oxygen demand [22] Also, argon of 25 to 80 vol% increased survival in rats exposed to normocapnic or hypercapnic hypoxia, and conversely potentiated oxygen-induced convulsions [22]. In addition, in rats exposed to non-lethal hypoxia (7 vol% oxygen for 1 hour), argon restored by ∼35% the mitochondrial respiratory enzyme activity [22], whose impairment following excitotoxic insults triggers neuronal death [23], [24]. Such a kind of argon-oxygen synergism could explain the neuroprotective effects of argon against hypoxia [14] and ischemia as reported in the present and previous studies [15], [17], [18]. Also, since previous data have shown redox modulation of the NMDA receptor with reduction inducing a potentiation and oxidation an inhibition of the NMDA receptor activity and glutamate-induced neuronal death [25], [26], the synergistic effect of argon on oxygen could explain its ability at reducing NMDA-induced neuronal death (a normoxic excitotoxic model). Indeed, in line with these data and the ability of argon at reducing NMDA-induced neuronal death, intrainsult oxygen has been shown to have antiexcitotoxic effects and to reduce NMDA-induced neuronal death and intraneuronal calcium influx [27], a critical event known to play a major role in excitotoxic and ischemic brain damage [1]–[3].

In contrast with its beneficial effect at the cortical level, we found that postischemic argon did increase MCAO-induced subcortical brain damage and further failed to reduce MCAO-induced neurologic deficits. This latter effect is in line with previous data that have demonstrated that neuroprotection requires preserving 80–90% of both the cortex and the subcortical areas to provide significant neurologic recovery and further predicted that 30% of subcortical brain damage might be sufficient to suppress any voluntary motor behavior [28]. Physiologically, the striatum that is the main part of the subcortical brain areas that suffer MCAO-induced brain damage is well recognized to be difficult to protect due to its lack of collateral vasculature and dramatic reduction in cerebral blood flow and oxygen supply as compared to the cortex. In these conditions, the occurrence of argon-induced oxygen synergy would not be possible as it occurs in brain slices exposed to OGD, where oxygen diffusion exists at the air-saline solution interface, in the cortex of rats subjected to MCAO-induced ischemia, where residual oxygen exists due to collateral vasculature, and in the striatum of rats subjected to NMDA injection that is a normoxic model of excitotoxic insult. Also, in contrast with the hypothermia-mediated neuroprotective effects of helium shown to result from the specific heat of helium that is higher than that of air [29], we found that argon as it could be expected from its specific heat that is half that of air produces mild hyperthermia, a condition well known to worsen ischemic brain damage that could have affected particularly the yet dramatically suffering subcortical brain areas as compared to the cortex. This latter possibility is in line with previous investigations that have shown that, in contrast with the cortex, neuroprotection of the striatum is tenuous and can be no longer obtained when time-to-treatment with xenon increases from 1 to 2 h after induction of ischemia or when blood/gas solubility (that is inversely proportional to diffusion) increases from 0.12 to 0.46 as a function of whether xenon or nitrous oxide is used to provide neuroprotection [30]. Finally, if one consider that argon would act by modulating membrane proteins [31] and particularly by potentiating the γ-amino-butyric acid type A (GABA_A_) receptor-mediated inhibitory neurotransmission [32], the present finding that postischemic argon provides no improvement of neurologic outcome while reducing cortical but not subcortical brain damage is in good agreement with a recent clinical study that has reported – in contrast with histological studies in animal models [33], [34] – that administration after reperfusion of GABA_A_ agonists, but not of anti-cholinergic drugs, disrupts the neuronal plasticity that supports functional recovery in stroke patients and thereby reinduces clinical deficits after stroke [35]. In line with this latter study, it has to be noted that xenon that provides dramatic postischemic neuroprotection possesses efficient antagonistic properties at the cholinergic neurotransmission [36].

It is noteworthy that the lack of neuroprotective effect of argon on ischemia-induced subcortical brain damage reported herein opposes recent data that have demonstrated argon-induced subcortical (and cortical) neuroprotection [18]. Rather than an actual discrepancy (different results obtained from similar experimental conditions), it is likely with little doubt that this can be attributed to major differences in the protocol used. Particularly, administration of argon during ischemia [18] or after ischemia (in the present report) could have played a major role, since previous studies have shown the critical importance of the time inert gases are given, during or after ischemia, to obtain neuroprotection [7], [20]. Also, in contrast with the work by Ryang and colleagues [18] where intraischemic argon was given for 1 h through a face mask in anesthetized rats, in the present study postischemic argon was given for 3 h in freely-moving animals not controlled for temperature in an anesthesia box, an environmental condition shown to allow the effect of inert gases on body temperature to occur much more rapidly than using face mask [37]. Finally, whether neuroprotection by intraischemic argon together with 50 vol% oxygen (*vs* 25 vol% oxygen+25 vol% nitrogen in the present study) can be fully attributed to argon remains actually and highly questionable since intraischemic oxygen has been shown to reduce ischemic brain damage both through anti-excitotoxic and thrombolytic processes (David et al., unpublished data).

The present study has possible weaknesses that must be discussed. First, although there is no real alternative to use an anesthetic such as isoflurane to induce transient brain ischemia, the impact of isoflurane administration in combination with 100 vol% oxygen on the findings of this study must be examined. Indeed, since isoflurane is known as a neuroprotectant preconditioning agent in the adult but not in the newborn rat [38]–[43], it is possible that residual isoflurane could have contributed at least partly to provide neuroprotection against MCAO-induced cortical brain damage. Conversely, it is possible that residual isoflurane could have contributed to increase MCAO-induced subcortical brain damage by producing apoptosis, an adverse effect of isoflurane yet demonstrated when given in combination with nitrous oxide but not alone [38], [41]. Likewise, since oxygen has been shown either to reduce or to increase excitotoxic and/or ischemic brain damage [27], [44], [45], it is possible that the use of 100 vol% oxygen to induce isoflurane anesthesia could have contributed also to reduce cortical brain damage and/or to increase subcortical brain damage. However, these possibilities are unlikely to be true since in the present study (i) the total duration of surgery was no more than 25 minutes, thereby allowing rapid isoflurane and oxygen desaturation from the rat's body and brain and thereby the virtual absence of residual isoflurane and oxygen during the period of treatment with argon [46]; (ii) isoflurane was never given in combination with argon. Support for this are previous data with the same protocol that have shown that xenon, in contrast with what found in the present study with argon, provides neuroprotection both at the cortical and subcortical levels [6]. Second, in this study, cerebral blood flow was not measured throughout MCAO since we wanted to shorten anesthesia to limit isoflurane effects. However, this should not have biased our results since postischemic argon obviously induces neuroprotective and adverse effects at the cortical and subcortical level respectively. Indeed, it is unlikely that such a dichotomic response could be attributed to a technical problem of occlusion/reperfusion, which if such should have biased data in the same way at the cortical and subcortical level.

In conclusion, our results do not support that postischemic argon could be an efficient alternative to xenon, shown to provide dramatic postischemic neuroprotection. However, given its antiexcitotoxic effect, it is possible that argon could be useful for treating other brain insults, such as brain trauma.

## Materials and Methods

### Animals

All animal-use procedures were examined by a local ethic committee (Cyceron, Caen, France) in accordance with the European Communities Council Directive for the use of animals in biomedical experimentation (Declaration of Helsinki), and approved with permit number 14–27. Adult male Sprague-Dawley rats (Janvier, Le Genest Saint-Isle, France) weighing 250 to 280 g were housed with food and water ad libitum by group of six (before surgery) or individually (after surgery) at a room temperature of 21±0.5°C with lights on from 8:00 pm to 8:00 am.

### *Ex vivo* OGD studies

OGD studies were performed as detailed previously [6]. Coronal brain slices of 400 µM thickness including the striatum were cut and allowed to recover at room temperature for 45 min in freshly prepared oxygenated artificial cerebrospinal fluid (aCSF). Cell injury was assessed by performing LDH activity assay. Brain slices were incubated individually at 36±0.5°C in oxygenated aCSF. After stabilisation of LDH release, the incubation aCSF was renewed and the slices were incubated for 1 h to allow recording basal LDH levels. Sham slices were incubated for an additional 20-min period in the same conditions. Brain slices subjected to OGD were incubated in a glucose-free solution continuously saturated with 100 vol% nitrogen. Then, to mimic reperfusion and treatment, the medium was replaced in all groups with aCSF continuously saturated with: (i) medical air; or (ii) argon of 25 vol% to 75 vol% (with the remainder being oxygen at 25 vol% completed with nitrogen when necessary). During the 3-h “reperfusion” period, aCSF was renewed every 1 h to obtain samples and to assess the kinetics of LDH release as a percentage change from LDH control value before OGD. The number of animals and slices per group was N = 4 and n = 23–27, respectively.

### *In vivo* NMDA studies

NMDA studies were performed aas detailed previously [6]. Briefly, rats were anesthetised with 1.5% halothane in oxygen alone, mounted on a stereotaxic apparatus, allowed breathing spontaneously, and maintained normothermic at 37±0.5°C. Rats were given 70 nmol NMDA in 1 µL PBS (pH 7.4) into the right striatum. Total duration of surgery was no more than 10 min, thereby allowing rapid halothane and oxygen desaturation from the rat's body, and the virtual absence of residual halothane and oxygen during post-NMDA treatment with medical air or argon [46]. The animals waked up in their home cage after about 10 min, with free access to food and water. Sixty minutes after injection of NMDA, the rats were treated as described below with either (i) medical air (controls); or (ii) argon of 25 vol% to 75 vol% (with the remainder being 25 vol% oxygen completed with nitrogen when necessary). The number of rats per group was as follows: 75 vol% argon, n = 7; 50 vol% argon, n = 8; 37.5 vol% argon, n = 8; 25 vol% argon, n = 8; 15 vol% argon, n = 8; NMDA control group, n = 11. Sham rats were given an intrastriatal saline solution and medical air.

### *In vivo* MCAO studies

MCAO studies were performed as described in details previously [6]. Briefly, rats were subjected to transient cerebral ischemia for 60 min by the intraluminal MCAO method using a nylon thread. Rats were anesthetised with 1.5% isoflurane in oxygen alone, allowed breathing spontaneously, and maintained normothermic at 37.5°C±0.5°C. Total duration of surgery was no more than 25 min, thereby allowing rapid isoflurane and oxygen desaturation from the rat's body, and little residual isoflurane and oxygen during the MCAO period [46]. After surgery, rats were allowed moving freely in their home wages with free access to food and water. Sixty minutes later, the nylon was removed under a short isoflurane anesthesia of less than 10 min duration, thereby allowing rapid isoflurane and oxygen desaturation from the rat's body, and the virtual absence of residual isoflurane and oxygen during the rats' reperfusion and 3-h post-MCAO treatment with either medical air (MCAO controls, n = 14) or argon at 50 vol% (n = 14) [46]. Treatment with medical air or argon at 50 vol% was performed according to a blinded procedure in a closed chamber at a flow rate of 1 chamber volume per minute, a condition that allowed maintaining carbon dioxide less than 0.03%. No analgesic treatment was given during recovery. Physiological parameters were recorded immediately before and immediately after treatment with medical air or argon at 50 vol%. Sham-treated rats (n = 6) were subjected to the same surgical protocol than controls and argon-treated rats, but not to ischemia, and exposed to medical air for 3 h.

The rats' neurologic outcome was assessed by recording the animals' motor coordination and rearing activity as detailed previously [6]. Motor coordination was quantified in individual non-motorized activity wheels equipped with 1/8 rotation sensors; rearing activity was quantified in individual activity cages equipped with infrared beams (Imetronic, Pessac, France). Beam interruptions and rotation signals were detected and recorded on a computer. Rats were familiarized with the activity cages and wheels. Then, on day 2 before MCAO (−48 h), and on day 1 (24 h) and day 2 (48 h) after MCAO, the rats were placed again and recorded for 15 min in the activity cages and the activity wheels. Post-MCAO scores of motor coordination and rearing activity were expressed as a percentage of pre-MCAO scores on day 2 before MCAO using each rat as its own control, and averaged to obtain a global score of neurologic outcome expressed as a percentage of that recorded in sham rats.

### Histologic analysis

Fifty hours after MCAO or NMDA injection, coronal brain sections (20 µm) were cryostat-cut, stained with thionin, digitized on a computer and analysed using an image analyzer (ImageJ® software, Scion corp., USA). The infarction volume was calculated by integration over the whole brain of the infarcted surfaces delineated by the pallor of staining compared to the surrounding healthy tissue, corrected for tissue oedema when needed (MCAO-induced ischemia) by calculating and dividing volume by the ipsilateral/contralateral brain hemispheres ratio, and expressed in mm^3^.

### Gas treatment

Oxygen, nitrogen and argon of medical grade were bought from Messer and Air Liquide. Gas mixtures containing 75 vol% nitrogen and 25 vol% oxygen (medical air), or argon of 15 vol% to 75 vol% (with the remainder being oxygen at 25 vol% completed with nitrogen when necessary) were obtained using calibrated flowmeters and gas analysers.

### Statistical analysis

Data on OGD-induced LDH release (n>20) are expressed as the mean ± the standard error to the mean (SEM) and were analysed using one-way parametric ANOVA and post-hoc unpaired Student t-test. Data on MCAO-induced brain damage (n<20) are expressed as the median value ± the 25–75^th^ percentiles and were analysed using the Kruskall-Wallis nonparametric ANOVA and/or post-hoc Mann-Whitney unpaired U-test. The number of animals per group for the *in vivo* studies was assessed through power analysis through online software (www.dssresearch.com/toolkit/sscalc/size_a2.asp). Because one objective of the study was to assess whether or not argon could be a cost-efficient alternative to xenon, the number of rats was calculated with the following variables from a previous study from our laboratory [6] with xenon at 50 vol%: mean ± standard deviation (SD) for ischemic brain damage volume in control group = 218±60 mm^3^; mean ± SD for ischemic brain damage volume in xenon-treated group = 59±28 mm^3^; α error level (confidence level) = 0.05; β error level (statistical power) = 0.01.
